# Patients’ Perceptions of Using Cold Plunge Therapy to Treat opioid use disorder

**DOI:** 10.1192/j.eurpsy.2026.10868

**Published:** 2026-07-31

**Authors:** L. Li

**Affiliations:** Psychiatry and Behavioral Neurobiology, University of Alabama at Birmingham, Birmingham, United States

## Abstract

**Introduction:**

Evidence-based treatment for opioid use disorder (OUD) is medications for OUD (MOUD), however, less than 25% of patients are taking MOUD. Possible reasons include stigma and skepticism. So, testing novel treatments to complement MOUD is essential. One potential treatment is cold plunge therapy (CPT), a brief, whole-body immersion in water <60°F, which. could be a potential complementary treatment for OUD. However, it is unknown if patients with OUD would be willing to try CPT as a complementary, nonpharmacological therapy.

**Objectives:**

We surveyed a sample of patients with OUD to assess the acceptability and feasibility of using CPT.

**Methods:**

We developed and administered a 16-item survey to 80 patients with OUD at the University of Alabama at Birmingham University Hospital, from April 1 to June 30, 2025. Eligible participants were identified using electronic medical records to find patients who were greater than 19 years old and had an OUD diagnosis (F11.X). Questions included gauging patient’s comfort discussing their OUD with friends and family, ease of access to buprenorphine prescriptions, and preferences for non-pharmacological treatment options such as physical activity, diet, sleep, prayer, and attending meetings. The survey was completed either by scanning a Qualtrics QR code or answering on paper anonymously, and descriptive statistics were calculated using SPSS, version 29.

**Results:**

Among 80 respondents, most were male (55%), white (75%), 35-44 years old (45%). About a third of respondents were from each of the three enrollment settings. On a 5-point Likert Scale, most respondents reported using illicit substances “A lot” to make them feel better physically (63.7%) or psychologically (67.5%). Participants also reported “Agree” (40%) or “Strongly Agree” (32.5%) regarding comfort discussing their OUD with family and friends, and “Strongly Agree” (58.8%) for their comfort seeking medical care for OUD treatment. Participants also indicated a willingness to participate in non-pharmacological treatment options (53.8%), including CPT (61.3%). The major barriers to considering CPT were unfamiliarity with CPT protocols, access to a cold-water tub, and/or discomfort with cold water. Most respondents (43.8%) stated they would prefer non-pharmacological treatment options, and 33.8% of respondents favored a combination of MAT and non-pharmacological treatment options.

**Conclusions:**

Patients with OUD, regardless of treatment setting, were agreeable to consider trying nonpharmacological treatments, including CPT, especially to complement MOUD.

**Disclosure of Interest:**

None Declared

